# Mitochondrial genomes of ancient bowhead whales (*Balaena mysticetus*) from Svalbard

**DOI:** 10.1080/23802359.2019.1693284

**Published:** 2019-11-22

**Authors:** Joost Grond, Magdalena Płecha, Christoph Hahn, Øystein Wiig, Lutz Bachmann

**Affiliations:** a Natural History Museum, University of Oslo, Oslo, Norway;; b Department of Molecular Phylogenetics and Evolution, Faculty of Biology, Biological and Chemical Research Center, University of Warsaw, Warsaw, Poland;; c Institute of Biology, University of Graz, Graz, Austria

**Keywords:** Ancient DNA, baleen whales, mitogenomics, Spitsbergen, Svalbard

## Abstract

The endangered Spitsbergen stock of bowhead whales (*Balaena mysticetus*) has once been large with up to estimated 100,000 individuals. Genetic diversity of the extant Spitsbergen stock is unknown. We present 10 complete mitochondrial genomes of heterochronous ancient bowhead whale samples from Svalbard (^14^C age estimate range: 215–8885 years) obtained via NGS of total genomic DNA extracts. The ten mitogenomes differed by nucleotide substitutions and/or indels, and there was a total of 160 variable positions. The average nucleotide diversity was *π* = 0.0029. There was no statistically significant correlation between genetic divergence and time.

## Introduction

Bowhead whales (*Balaena mysticetus*) are the only baleen whale species that occur year-round in Arctic and subarctic regions. The Spitsbergen stock is distributed in the Greenland Sea and the northern Barents Sea, and is today classified as being Endangered by The International Union of Conservation of Nature (IUCN) (Cooke and Reeves 2018). Estimates of the stock size before the onset of extensive hunting in the early 17th century range from 25,000 to 100,000 individuals (Allen and Keay 2006). A recent stock size estimate that took into account combined helicopter- and ship-based line transect surveys was roughly 350 individuals (Vacquié-Garcia et al. 2017).

Only two studies have hitherto used genetic approaches on Spitsbergen stock bowhead whales. First, based on D-loop sequences Borge et al. (2007) assessed the mitochondrial haplotype diversity in ancient bone remains from 99 bowhead whales and reported a very high genetic diversity. The most abundant mitochondrial haplotypes were the same as those in the extant Bering/Chukchi/Beaufort Seas (BCB) stock. Second, Nyhus et al. (2016) provided full mitochondrial genomes from eight contemporary samples collected in the Fram Strait during ship-based surveys in 2006 and 2010. Here we provide mitochondrial genomes of ancient Spitsbergen stock samples for an improved understanding of the mitogenomic diversity of this bowhead whale population.

## Materials and methods

Bones from the 10 bowhead whales included in this study were collected at Svalbard during 1976–1997 for the purpose of dating raised beaches (for details see Borge et al. 2007). The ^14^C age of the bones ranged from 225 to 8885 years. Total genomic DNA was extracted in a laboratory specifically dedicated to ancient DNA work at the Natural History Museum Oslo as described by Borge et al. (2007). Preparation of paired-end libraries, and analysis on an Illumina HiSeq 2000 was outsourced to StarSEQ GmbH, Mainz, Germany.

The 100 bp raw reads of the bowhead whale samples were quality trimmed (TrimGalore 0.3.3., https://www.bioinformatics.babraham.ac.uk/projects/trim_galore/), and the mitochondrial genomes were assembled using MITObim 1.8 (Hahn et al. 2013) with GenBank entry AJ554051 as bait sequence. Both programs were used with default parameters. The assembled mitochondrial genome sequences and the raw mitochondrial reads (as identified by MITObim) were deposited in GenBank and NCBI's Sequence Read Archive (SRA; Bioproject: PRJNA558634), respectively. Accessions are listed in Table 1.

**Table 1.** Spitsbergen stock bowhead whale samples included in this study for NGS of complete mitochondrial genomes.

Sample ID*	Sample ID**	^14^CAge	Locality***	Latitude N	Longitude E	mt-reads	Coverage (x)	D-loop haplotype	GenBank accession no	SRA accession no
NHMO-DMA-43194	11062	215	Edgeøya	77°40′00′′	22°30′00′′	8460	50.0	BWS13**	MN124685	SRR9937203
NHMO-DMA-43195	11063	325	Edgeøya	77°40′00′′	22°30′00′′	21,840	126.6	BWS12**	MN145937	SRR9937202
NHMO-DMA-43197	11065	325	Lurøya	77°00′00′′	21°55′00′′	2332	14.5	BWS1**	MN145938	SRR9937201
NHMO-DMA-43191	11059	370	Kong Ludvigøyane	77°10′00′′	21°00′00′′	1181	7.8	BWS6**	MN145939	SRR9937200
PMO 234.573		375	Kaffiøyra	78°38′00′′	11°55′00′′	1579	10.2	BWS20**	MN159080	SRR9937199
PMO 234.423	1985–52	405	Kaffiøyra	78°38′00′′	11°55′00′′	1930	12.2	BWS19**	MN159085	SRR9937198
NHMO-DMA-43206	11074	415	Mohnbukta	78°19′00′′	18°55′00′′	6284	37.1	BWS11**	MN159084	SRR9937197
PMO 234.525		990	Hornsund	76°56′00′′	15°46′00′′	2297	14.3	BWS59	MN159083	SRR9937196
PMO 234.429	1981–89	7380	Dicksonfjorden	78°43′00′′	15°29′00′′	1465	9.6	BWS1****	MN159082	SRR9937205
PMO 234.522		8885	Sassenfjorden	78°26′00′′	16°31′00′′	2114	13.2	BWS1**	MN159081	SRR9937204

^*^ all bone samples deposited at Natural History Museum, University of Oslo, Norway.

^**^ according to Borge et al. 2007.

^***^ all located on Svalbard.

^****^ Borge et al. 2007 reported BWS25.

The mitochondrial genome sequences were aligned using the ClustalW option in MEGA 7 (Kumar et al. 2016). Maximum Likelihood analyses were carried out in PHYML 3.0 (Guindon et al. 2010) applying the HKY85 + G + I model as selected by the program’s SMS option.

The relationship between genetic divergence and time of the heterochronous bowhead whale mitogenomes was explored with TempEst v. 1.5.3. (Rambaut et al. 2016) using the ‘best-fitting root’ option. The required phylogenetic tree constructed without assuming a molecular clock was generated using IQ-TREE v. 1.6.12 (Nguyen et al. 2015).

## Results and discussion

The assembled mitochondrial genomes were 16,389–16,391 bp long; indels were only observed in the 12S gene (sample PMO 234.423) or the D-loop region. Nine samples have been used earlier for mitochondrial haplotype determination through a 453 bp stretch of the D-loop region (Borge et al. 2007; positions 15,475–15,927 in the alignment of supplemental material S1). The respective regions of the assembled mitogenomes matched the previously reported haplotypes for these samples with the exception of sample PMO 234.429, which had a different haplotype (BWS25) assigned by Borge et al. (2007). In addition, one new D-loop based haplotype (BWS59) was found in sample PMO 234.525. Haplotype BWS1, previously identified as the most common haplotype in ancient bowhead whale bones from Svalbard was found in three samples; however, the respective three mitogenome sequences were not identical and differed by nucleotide substitutions elsewhere in the sequence.

Among the 10 ancient bowhead whale mitogenomes there were 160 variable sites (154 transitions, 4 transversion, and 3 indels). An extended alignment including the mitogenomes available in GenBank (AP00672 from the Okhotsk Sea, AJ554051 of unknown origin, and KY026766, KY026773, and KY026772 from contemporary Svalbard stock samples) resulted in 206 variable sites (185 transitions, 14 transversion, and 7 indels) across the 15 haplotypes. In this expanded dataset, 143 substitutions affect protein-coding regions (103 synonymous and 40 non-synonymous substitutions), 29 RNA genes, and 32 noncoding positions. Not surprisingly, the non-coding D-loop region was the most variable part of the mitochondrial genomes.

**Figure 1.**
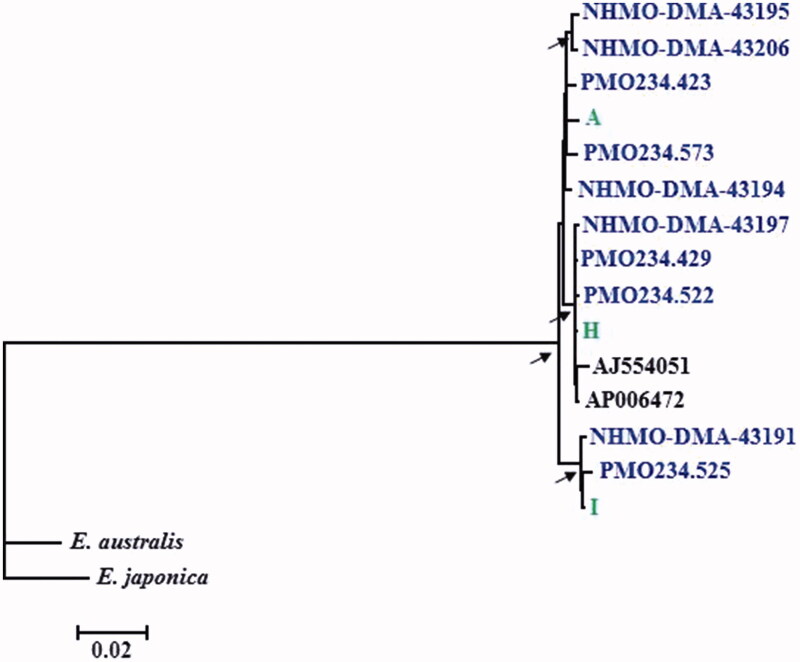
Maximum Likelihood tree of 15 mitochondrial genome sequences from 10 ancient (blue) and five contemporary bowhead whales including three from the current Spitsbergen stock (green). Mitogenome sequences of southern right whale (*Eubalaena australis*) and North Pacific right whale (*E. japonica*) were used as outgroups. Bootstrap support values were estimated from 1000 replicates, and those exceeding 90% are indicated by arrows at the respective nodes.

Average nucleotide diversity was *π* = 0.0029 for the 10 ancient Spitsbergen mitochondrial haplotypes. A maximum-likelihood phylogram (Figure 1) depicts the relationships of the ancient and contemporary mitochondrial genomes of Spitsbergen stock bowhead whales. There was no particular haplotype structure, and the ancient mitogenomes group together with the contemporary mitogenomes in several haplogroups. This is in line with previous reports on D-loop based analyses of ancient Spitsbergen stock (Borge et al. 2007) and contemporary Behring-Chukchi-Beaufort stock samples Rooney et al. 2001).

There was no statistically significant association between genetic divergence through time and sampling dates. For the Spitsbergen stock bowhead whale mitogenomes included in this study the correlation coefficient was 0.1135 (R^2^: 0.012883) with PMO 234.525 (above) and NHMO-DMA-43194 (below) deviating most from the ‘root-to-tip’ regression line.
